# Supplementary material for: Codelivery of HBx-siRNA and Plasmid Encoding IL-12 for Inhibition of Hepatitis B Virus and Reactivation of Antiviral Immunity
Source: Pharmaceutics. 2022 Jul 9;14(7):1439. doi: 10.3390/pharmaceutics14071439 (PMC9318813; doi:10.3390/pharmaceutics14071439)
Supplement: Supplementary file 1 [file pharmaceutics-14-01439-s001.zip › pharmaceutics-1782347-supplementary.pdf]

Supporting Information

Codelivery of HBx-siRNA and Plasmid Encoding IL-12 for Inhibition of Hepatitis B Virus and Reactivation of Antiviral Immunity

Yan Mu [1,2,3], Xiao-He Ren [4], Di Han [4], Ying-Ying Guan [1,2,3], Pei-Ling Liu [1,2,3], Si-Xue Cheng [4] and Hong Liu [1,2,3,]*

[1] National & Local Joint Engineering Research Center of High-Throughput Drug Screening Technology, College of Life Sciences, Hubei University, Wuhan 430062, China

[2] Key Laboratory of Biotechnology of Chinese Traditional Medicine of Hubei Province, College of Life Sciences, Hubei University, Wuhan 430062, China

[3] Hubei Collaborative Innovation Center for Green Transformation of Bio-Resources, College of Life Sciences, Hubei University, Wuhan 430062, China

[4] Key Laboratory of Biomedical Polymers of Ministry of Education, Department of Chemistry, Wuhan University, Wuhan 430072, China

* Correspondence: liuhong@hubu.edu.cn

Supplementary Methods

Characterizations of siRNA and/or pIL-12 Loaded Complexes

The as-prepared solution containing siRNA and/or pIL-12 loaded complexes was diluted with deionized water to a total volume of 1 mL, and then subjected to size and ζ-potential mesaurements by a Zetasizer (Nano ZS, Malvern Instruments).

The morphology of the complexes was visualized by transmission electron microscopy (TEM) (Talos L120C G2) at 120 kV. The samples were stained with a phosphotungstic acid aqueous solution.

In Vitro Cytotoxicity Assay

1×10^4 cells were seeded in the well of a 96-well plate and incubated with 100 μL of culture medium containing 10% FBS at 37°C. After 24 h, the culture medium was replaced with 100 μL of fresh culture medium containing siRNA and/or pIL-12 loaded complexes. The cells were incubated for 48 h. Then 10 μL of MTT (10 mg/mL) was added in each well, followed by incubation for 4 h at 37°C, finally the supernatant was removed carefully and 150 μL of DMSO was added dissolving the formazan crystals produced by cells. The microplate reader (infinite 200Pro) was used to measure the OD value at 570 nm. The cell viability was calculated as

cell viability = $OD_{treated} - OD_{blank} / OD_{control} - OD_{blank} \times 100\%$,

in which $OD_{treated}$ was measured after the cells were treated by particular agents, $OD_{control}$ was measured for the cells without treatment, and OD_{blank} was measured without the presence of cells.

In Vitro Cytokine Assay

1×10^5 cells were seeded in per well of 12-well plate with 1 mL of culture medium and incubated at 37°C for 24 h. After that, the medium was replaced with 1 mL of fresh culture medium containing siRNA and/or pIL-12 loaded complexes. Unless otherwise mentioned, the cells were co-incubated with siRNA and/or pIL-12 loaded complexes at siRNA concentration of 60 pmol/mL for HepG2.215 cells or 300 pmol/mL for J774A.1 cells, and pIL-12 concentration of 1 μg/mL for HepG2.215 cells or 5 μg/mL for J774A.1 cells for 48 h. After that, the culture supernatant was collected and then subjected to ELISA according to the manufacturer's protocol.

qPCR Assay

1×10^5 cells were seeded in per well of 12-well plate with 1 mL of culture medium and incubated at 37°C for 24 h. After that, the medium was replaced with 1 mL of fresh culture medium containing siRNA and/or pIL-12 loaded complexes. The cells were co-incubated with siRNA and/or pIL-12 loaded complexes at siRNA concentration of 60 pmol/mL for HepG2.215 cells or 300 pmol/mL for J774A.1 cells, and pIL-12 concentration of 1 μg/mL for HepG2.215 cells or 5 μg/mL for J774A.1 cells for 48 h. After that, the cells were carefully washed, and then collected for qPCR analysisas detailed below.

The total RNA was extracted with a High Pure RNA Isolation Kit (Invitrogen). An EntiLink™ 1st Strand cDNA Synthesis Kit (ELK Biotech) was used for the first cDNA strand synthesized, and then qPCR was performed on a StepOne Real-Time PCR system (Life Technologies) with EnTurbo™ SYBR Green PCR SuperMix (ELK Biotech). The relative RNA levels were measured by the $2^{-\Delta\Delta Ct}$ method.

Western Blot Analysis

2×10^5 cells were seeded in per well of 6-well plate with 2 mL of culture medium and incubated at 37°C for 24 h. After that, the medium was replaced with 2 mL of fresh culture medium containing siRNA and/or pIL-12 loaded complexes. The cells were co-incubated with siRNA and/or pIL-12 loaded complexes at siRNA concentration of 60 pmol/mL for HepG2.215 cells or 300 pmol/mL for J774A.1 cells, and pIL-12 concentration of 1 μg/mL for HepG2.215 cells or 5 μg/mL for J774A.1 cells for 48 h. After that, the cells were carefully washed, and then collected for western blot analysis as detailed below.

Total protein extracts were subjected to SDS-polyacrylamide gel electrophoresis (PAGE). After electrophoresis, the proteins were transferred to PVDF membranes (Millipore). To block non-specific binding sites, the membranes were incubated with the primary antibody, rabbit polyclonal anti-P170 antibody (1/1000 dilution), overnight at 4 ºC. After washing, the membranes were sequentially incubated with the secondary antibody for 0.5 h.

Supplementary data

Table S1. Amounts of components for the preparation of siRNA and/or pIL-12 loaded complexes.

Sample	siRNA (pmol)	pIL-12 (μg)	Lipofectaime 2000 (μg)
siRNA@lipo	60	-	2
pIL-12@lipo	-	1	2
siRNA/pIL-12@lipo	60	1	2
siRNA@lipo*	300	-	4
pIL-12@lipo*	-	5	4
siRNA/pIL-12@lipo*	300	5	4

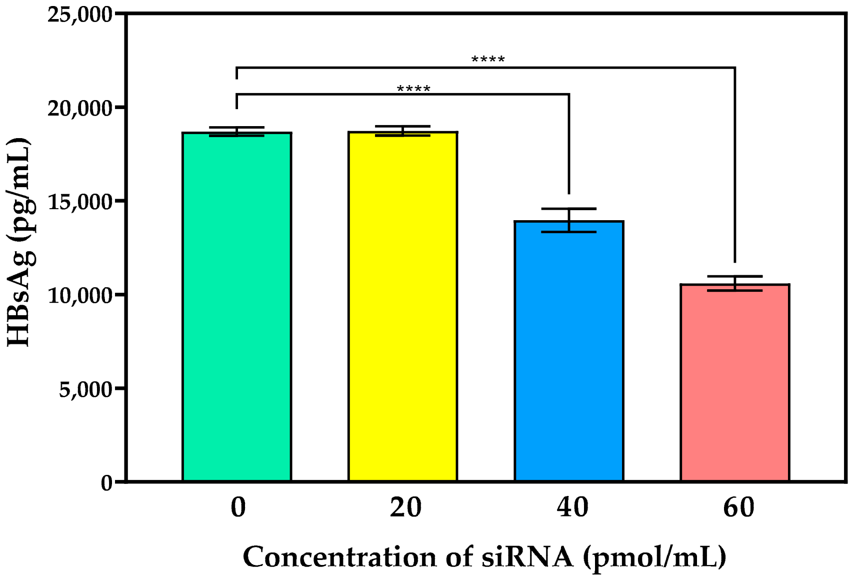

Figure S1. The concentration of HBsAg in the culture supernatants of HepG2.215 cells treated by siRNA@lipo with different concentrations. ****p<0.0001. n=3.

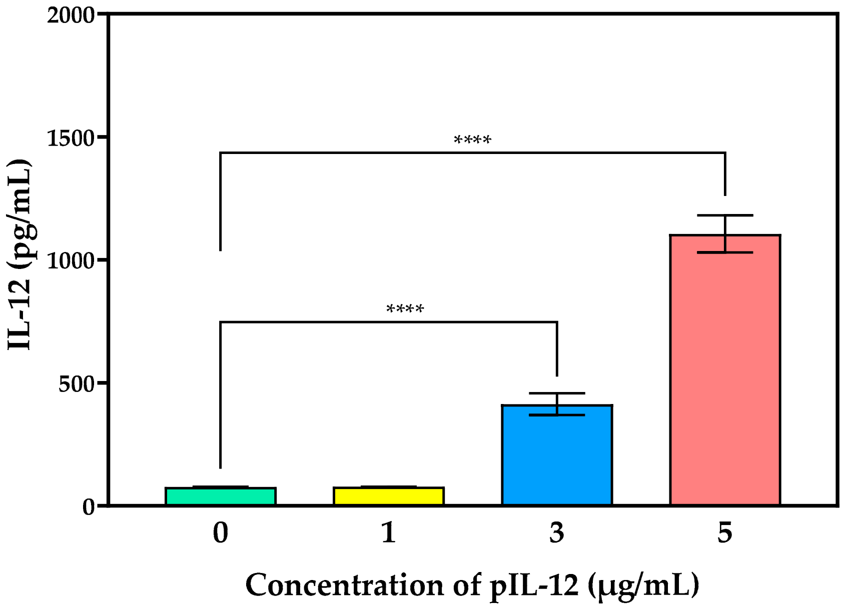

Figure S2. The concentration of IL-12 in the culture supernatants of J774A.1 cells treated by pIL-12@lipo with different concentrations. ****$p<0.0001$. $n=3$

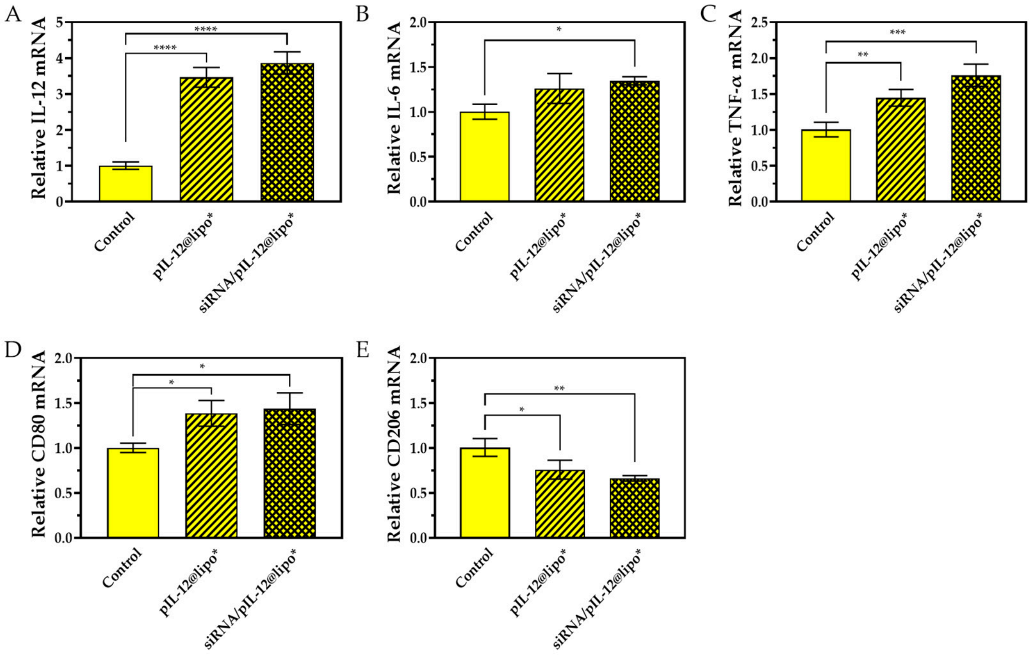

Figure S3. The mRNA levels of IL-12 (A), IL-6 (B), TNF-α (C), CD80 (D) and CD206 (E) in J774A.1 cells treated with pIL-12 containing complexes for 48 h at the siRNA concentration of 300 pmol/mL and/or the pIL-12 concentration of 5 μg/mL. J774A.1 cells without treatment were used as a control. *$p<0.05$. **$p<0.01$. ***$p<0.001$. ****$p<0.0001$. n=3
